# The Medical Society

**Published:** 1897-03-13

**Authors:** 


### Medical Progress and Hospital Clinics.

[*The Editor will be glad to receive offers of co-operation and contributions from members of the profession. All letters should be addressed to* The Editor, at the Office, 28 & 29, Southampton Street, Strand, London, W.C.]

### THE MEDICAL SOCIETY.

At the meeting last Monday, Mr. C. B. Lockwood, after giving a review of the operation for excision of the knee, described a new procedure which he had devised in the hope of obviating some of the difficulties, and of obtaining a more perfect result in those cases in which excision of the knee was deemed desirable. Different cases required different treatment, and during the past ten years he had treated tuberculous arthritis of the knee by rest and local applications, by drainage, by arthrectomy, by the ordinary excision, and by the operation now to be described. This last was done upon cases of progressive tuberculous arthritis with pulpy synovial membrane and ulceration of cartilage, but with little caries or displacement of the ends of the bones. Before deciding to operate, every effort had been made to allay the disease by rest and local applications, but it was important not to delay until sinuses had formed.

The operation described by Mr. Lockwood aimed at the retention of the patella and its fixation to both the femur and the tibia, the retention of as much as possible of the crucial ligaments, and the division of the bones in such a manner that the upper end of the tibia should form a wedge which should lock into a furrow made between the remains of the two condyles of the femur. The operation might be described as follows: The knee being bent at half a right angle, a slightly-curved incision is carried across the centre of the ligamentum patellæ from one tuberosity of the femur to the other. The ligamentum patellæ is then divided and the joint opened. The joint is then bent to a right angle, and the extent of the disease is ascertained. Then the synovial membrane is removed with knife and scissors as carefully as if it were full of malignant disease. The removal of the part behind the condyles is conveniently left until the femur has been sawn. Healthy ligaments are to be preserved, but it certainly is very hard to remove all the synovial membrane or the necessary amount of bone without cutting the anterior crucial ligament. This one, however, is not so important as the other, which is chiefly concerned in preventing the tibia falling backwards after the operation. Nevertheless, it is better to destroy both ligaments than to leave any synovial membrane behind. In the treatment of the bones first the articular surface of the patella is sawn off, and, if necessary, its cancellous tissue gouged. Then the trochlear surface of the femur is sawn off, leaving a flat surface, upon which the patella fits. The femur is next raised vertically, and the greater part of its articular surface is taken away by two saw cuts, which begin at the convexity of the condyles at the edge of the articular cartilage. These saw cuts are carried obliquely into the intercondyloid notch of the femur until they converge, entering the notch at the edge of the articular cartilages, and below the attachments of the crucial ligaments, the lower part of the attachments of which, however, cannot always be saved. The antero-posterior plane of these cuts should be at right angles to the axis of the femur. The remaining articular cartilage is then removed. The tibia is then made to fit the antero-posterior groove thus made in the femur. With a narrow saw two oblique cuts are carried upwards from the epiphisial line, commencing one at the inner and the other at the outer side of the tibia and converging at the base of the spinous process. In doing this it is quite feasible to remove the articular cartilages without destroying the lower attachments of the crucial ligaments. A piece is then sawn vertically off the front of the tibia for the patella to lie upon, and the operation is finished by sewing together the divided ligamentum patellæ with buried silk sutures and closing the wound. The advantages claimed for the operation are that it is much easier by it to keep the cut surfaces of the bone in contact and at rest; neither lateral movement nor rotation is possible; under favourable circumstances no falling back of the tibia can occur; the growth of the epiphyses is less interfered with; and finally the ultimate result is a very firm synostosis with hardly any tendency to flexion; while to gain these benefits the patient runs no risks beyond those of the usual operation.

In the discussion which followed some doubt was thrown upon the great importance of retaining the ligaments. It was pointed out that however the bones were cut a certain shortening must result, and that in consequence of this the ligaments must be so slack after the operation as to be of but small, if any, use in maintaining the proper relations between the tibia and the femur.

Mr. Marmaduke Sheild advocated the use of a Howse's splint, while Mr. Boyd thought that the splint of all others was a Thomas's knee splint; but it was clear that the object of both was the same, viz., the maintenance of immobility of the limb, which, of course, was one of the things claimed by Mr. Lockwood for his operation.

Under the title of "Some Cases of Intestinal Neurosis," Mr. Clinton Dent read a paper in which he urged that, notwithstanding all that had been said in favour of operative measures in such cases, it was not right to perform laparotomy on the chance of good resulting in some unexplained way, in cases in which the only diagnosis that could be arrived at was that of intestinal neurosis. To some, no doubt, this might seem a proposition which required but little argument in its favour; but it may be taken as a measure of the readiness to open the peritoneal cavity on the slightest provocation which exists at present, that the author of this paper almost apologised for the apparent reactionary nature of the suggestion which he was putting forward.

In the discussion which followed Mr. Marmaduke Sheild struck the proper keynote when he said that, in many cases similar to those which Mr. Dent had described, it was really impossible to be quite sure what was the nature of the case until an operation had been undertaken. It was not so much that it was good to operate in cases of intestinal neurosis, as that it was impossible by any ordinary means to make quite sure that nothing more than a neurosis existed; and he related a case which had been most carefully investigated both by himself and others, a case which was in every way suggestive, by the whole train of its symptoms, of being neurotic, a case in which it might almost be said that he operated against his better judgment, and yet in which, on laparotomy being performed, tuberculous nodules were found scattered over the peritoneum, and a tube was found tied down. The result was that the exhausting vomiting, which had been the prominent symptom, was at once relieved. The teaching of the case, however, was not that that operation for a pure neurosis was justifiable, but that till organic disease could be excluded with far greater certainty than at present could be done, it was impossible to say that any patient was suffering from nothing more than an intestinal neurosis.

